# Retraction: Medication regimen complexity and its association with adherence and blood pressure control among hypertensive patients at selected hospitals of South Gondar Zone: A hospital based cross sectional study

**DOI:** 10.1371/journal.pone.0282464

**Published:** 2023-06-23

**Authors:** The PLOS ONE Editors

After this article [1] was published, it came to light that the authors did not have permission to use the MMAS^®^-8 scale [2].

In addition, PLOS identified concerns about the article’s [1] study design and reporting. The MMAS^®^-8 scale was adapted and translated for this study, but the methods used for the adaptation and translation were not clearly reported. Furthermore, based on information PLOS received it does not appear as though the adapted and translated versions were rigorously evaluated as to their construct validity and reliability. The article reports that a “pretest” was done, but the details of this pretest are unclear and the article does not report the rationale for the low sample size used in this assessment.

The corresponding author provided the following responses to the study design concerns.

- The questionnaire was translated to Amharic and then back translated to English to evaluate the consistency.
- Data were collected using adherence questions reported in [3], and per the authors the reliability of the MMAS^®^-8 scale was assessed previously in [4, 5].
- A sample size of 5% was used for the pretest.
- For the main study’s sample size, the authors used “the magnitude of the problem as 50%” on the basis that no similar study had been conducted at the study sites previously.

The information received from the authors in post-publication discussions did not fully resolve the study design and permissions concerns. *PLOS ONE* considers that these are critical issues given the reliance of the study on the MMAS^®^-8 scale and community standards for survey tool development and validation.

In light of these issues, the *PLOS ONE* Editors retract this article. We regret that the concerns were not identified and addressed during the article’s pre-publication peer review.

The corresponding author stated that the authors do not agree with retraction. The other authors did not respond directly or could not be reached.

The article was republished at time of retraction to remove contents from the Methods, Results, Conclusions, and Supporting Information that directly relate to the MMAS^®^-8 work.

Please note that the MMAS^®^-8 scale (used in this study) was originally published in 2008 [2], but the copyright for the scale was registered by Donald E. Morisky on September 21, 2018 (U.S. Copyright Registration No. TX0008632533/2018-09-21). The MMAS^®^ trademark was also registered by Donald E. Morisky on January 29, 2019 (Reg. No. 5837374). Any reference to “MMAS” in this article [1] is for research fair use purposes.
